# Cauda equina cavernous malformation with nerve fascicle involvement and chronic bowel and urinary urgency: a case report

**DOI:** 10.3389/fsurg.2026.2004471

**Published:** 2026-10-22

**Authors:** Shumei Wang, Jifeng Shang

**Affiliations:** 1 Department of Neurosurgery, Shenzhen University General Hospital, Shenzhen, Guangdong, China; 2 Department of Neurosurgery, The University of Hong Kong-Shenzhen Hospital, Shenzhen, Guangdong, China

**Keywords:** case report, cauda equina, cavernous malformation, intradural extramedullary, nerve fascicles, urinary urgency

## Abstract

**Background:**

Cavernous malformations of the cauda equina can resemble nerve sheath tumors. Their relationship to functioning nerve fibers complicates surgical decision-making, and preserved motor strength alone may not capture postoperative morbidity.

**Case description:**

A 51-year-old woman presented with four years of low back discomfort, numbness of the right fourth and fifth toes, with occasional later numbness of the left fourth and fifth toes, and progressive bowel and urinary urgency without incontinence or weakness. Preoperative MRI demonstrated an approximately 11 × 10 × 15 mm intradural extramedullary nodule at L2, with mildly increased T1 signal, heterogeneous low T2 signal, and heterogeneous enhancement. A cavernous malformation and a neurogenic tumor were considered. At surgery, a gray-purple vascular lesion displaced the cauda equina roots, with a substantial nerve bundle traversing the mass. Most fascicles were separated; a small inseparable portion was coagulated and divided to permit complete excision. Reported motor and somatosensory evoked potentials remained within normal limits; pathology was labeled “cavernous hemangioma.” Postoperatively, bilateral hip pain with intermittent leg radiation improved with treatment, although mildly effortful urination was documented on postoperative day 7. At discharge on day 10, strength was grade 5 and no superficial sensory deficit was recorded. Postoperative MRI was obtained approximately four months after surgery. Longer-term autonomic outcomes were not documented.

**Conclusion:**

This case highlights the need to consider a cavernous malformation in the differential diagnosis of an L2 intradural mass and to report autonomic and pain outcomes separately from motor preservation after resection involving adherent nerve fascicles.

## 1 Introduction

Cavernous malformations of the cauda equina are uncommon vascular lesions that may present as circumscribed intradural extramedullary masses. In a surgical series of 10 patients, imaging appearances were variable and the peripheral hemosiderin ring was uncommon (1). A recent report also documented a thoracolumbar cavernous malformation initially interpreted as a schwannoma (2). Thus, recognition of a mass in this compartment does not reliably establish its histological identity.

Nerve root adherence introduces a further problem. Apostolakis and colleagues described a cavernoma requiring sacrifice of an adherent root to achieve complete removal (3), while another recent case involved a lesion among the cauda equina rootlets (4). We report a patient with prolonged bowel and urinary urgency and minimal objective motor impairment whose L2 lesion incorporated nerve fascicles. The educational focus is the relationship between diagnostic uncertainty, intraoperative fascicle preservation, and the interpretation of early postoperative symptoms. The report is organized using the CARE framework (5).

## 2 Case description

A 51-year-old woman was admitted with a four-year history of low back discomfort, numbness of the right fourth and fifth toes, and bowel and urinary urgency. Symptoms began in 2021 without an identified precipitating event. She reported neither incontinence nor lower-limb weakness. Over subsequent years, urgency and right toe numbness increased, and occasional numbness developed in the left fourth and fifth toes. Walking and other activity exacerbated symptoms, whereas bed rest provided gradual relief.

An outside MRI in December 2024 showed an approximately 12 × 14 mm left-sided intradural extramedullary lesion at the L2 vertebral level, considered likely benign. Associated findings included mild lumbar degeneration and an L5–S1 disc bulge. Her history included bilateral ovarian cyst excision. The clinical course is summarized in Table 1.

**Table 1:** Clinical timeline.

Time relative to surgery	Documented event
2021	Onset of low back discomfort, right fourth and fifth toe numbness, and bowel and urinary urgency; no incontinence or weakness.
December 2024	Outside MRI: L2 intradural extramedullary lesion, approximately 12 × 14 mm.
Day −2	Admission for progressive symptoms; mild right fourth and fifth toe sensory abnormality; occasional left fourth and fifth toe numbness was reported by admission.
Day −1	MRI: 11 × 10 × 15 mm L2 nodule; cavernous hemangioma versus neurogenic tumor. Postvoid residual: 1.3 mL.
Day 0	20 March 2025: bilateral L1 laminectomy and complete excision documented intraoperatively; small inseparable fascicle component divided; no monitoring alert reported.
Days 1–2	Preserved limb movement; catheter removed; hip pain and constipation treated.
Day 5	Final pathology: cavernous hemangioma.
Day 7	Improving hip pain; mild effortful urination; gross sensory and motor examinations normal.
Days 9–10	Sutures removed; discharged with grade 5 strength and healed incision.
Three months planned	Contrast-enhanced lumbar MRI advised at discharge.
Day 127	25 July 2025: postoperative lumbar MRI obtained; screenshots available.

Day 0 denotes surgery.

At admission, there was no scoliosis or focal lumbar midline or paraspinal tenderness. Two-point discrimination over the lateral right toes was mildly reduced compared with the opposite side. Other recorded superficial and deep sensory findings were unremarkable. Preoperative examination found no saddle sensory abnormality. Limb strength and tone were normal; patellar and Achilles reflexes were present. Ankle clonus and pathological reflexes were absent bilaterally. The preoperative postvoid residual volume was 1.3 mL. Electromyography of both lower limbs was normal.

### 2.1 Diagnostic assessment

Whole-spine MRI with and without contrast, performed one day before surgery, demonstrated a well-demarcated nodule at approximately the L2 vertebral level, measuring 11 × 10 × 15 mm. The radiology report described mildly increased T1-weighted signal, heterogeneous low T2-weighted signal, mildly increased diffusion-weighted signal with a reduced apparent diffusion coefficient signal, and heterogeneous enhancement. These are qualitative findings from the contemporaneous report; quantitative diffusion measurements were not available. Representative preoperative MRI screenshots obtained on 19 March 2025 are shown in Figure 1.

**Figure 1:**
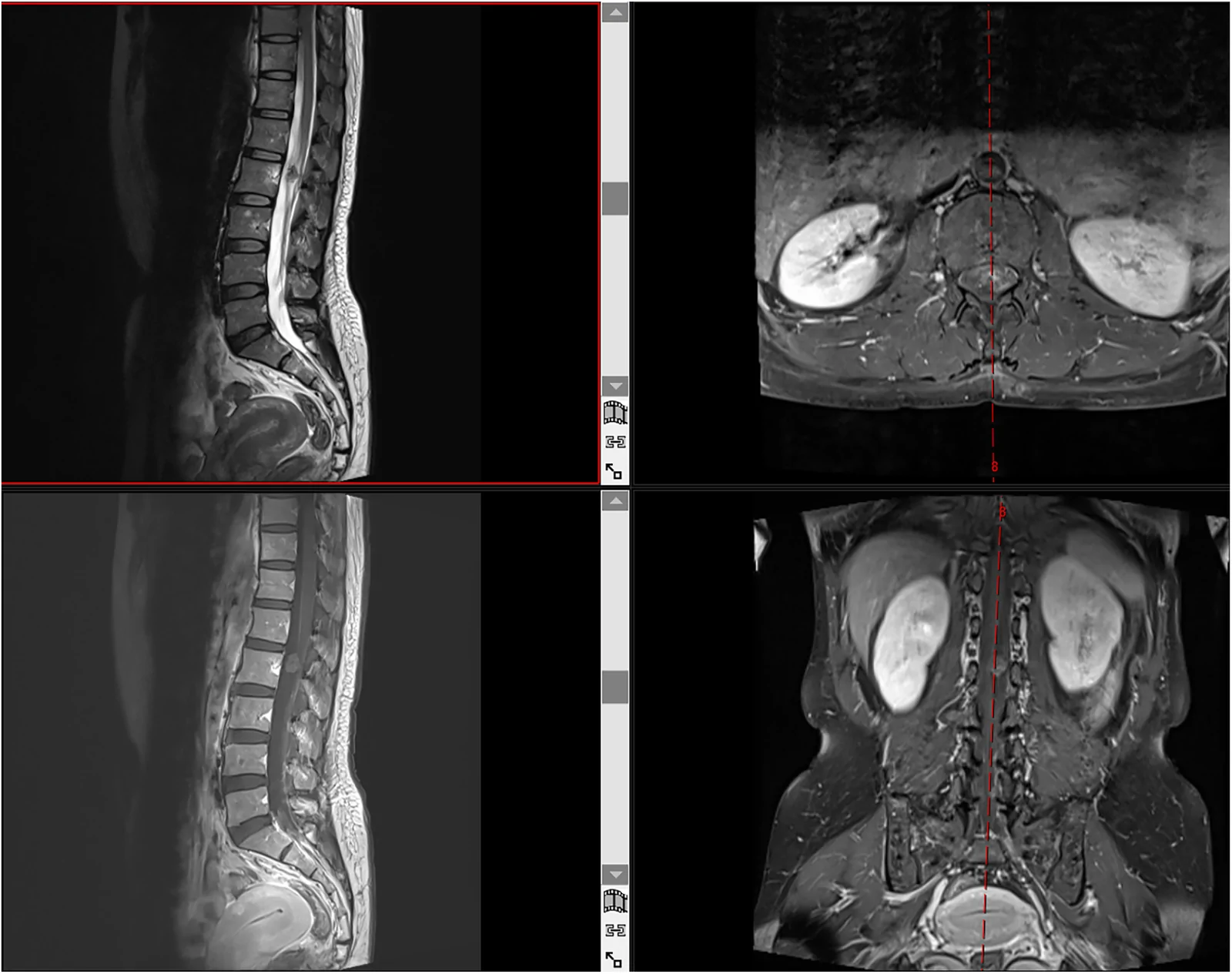
Preoperative lumbar MRI. Preoperative lumbar MRI obtained on 19 March 2025. The composite screenshot contains sagittal views on the left, an axial view at the upper right, and a coronal view at the lower right. These show an 11 × 10 × 15 mm intradural extramedullary nodule at L2, with mildly increased T1 signal, heterogeneous low T2 signal, and heterogeneous enhancement.

The radiologist considered recurrent chronic hemorrhage or proteinaceous contents and suggested a cavernous hemangioma versus a neurogenic tumor. The admission working diagnosis had been schwannoma. Chronic hemorrhage remained an imaging interpretation rather than a demonstrated sequence of bleeding events. There was no reported vertebral destruction. A small ventral T4 signal abnormality, visible principally on the fat-suppressed T2 sequence, was favored to represent cerebrospinal fluid flow artifact. It was not treated as a second cavernous malformation.

The decision to operate was based on the symptomatic intradural lesion and progressive complaints. The very small postvoid residual demonstrated effective emptying at the time of measurement, but did not characterize the mechanism of urgency. Formal urodynamic findings and standardized bowel or bladder symptom scores were unavailable.

### 2.2 Therapeutic intervention

Surgery was performed under general anesthesia on 20 March 2025. The patient was positioned prone, electrophysiological monitoring leads were connected, and the L2 level was localized using radiography. A posterior midline incision of approximately 6 cm was made. A bilateral L1 laminectomy was performed to expose the dura, with meticulous hemostasis throughout bony exposure. All intraspinal steps were performed under the operating microscope.

After longitudinal dural opening and arachnoid dissection, a gray-purple lesion with an intact capsule-like surface and multiple tortuous surface vessels was identified. The cauda equina roots were displaced laterally, and a relatively substantial nerve bundle passed through the lesion. The surgeons separated as many fascicles as possible. A small portion remained inseparable from the mass and was coagulated and divided, after which complete removal was documented. The intraoperative photograph shows the lobulated purple lesion and adjacent neural structures (Figure 2).

**Figure 2:**
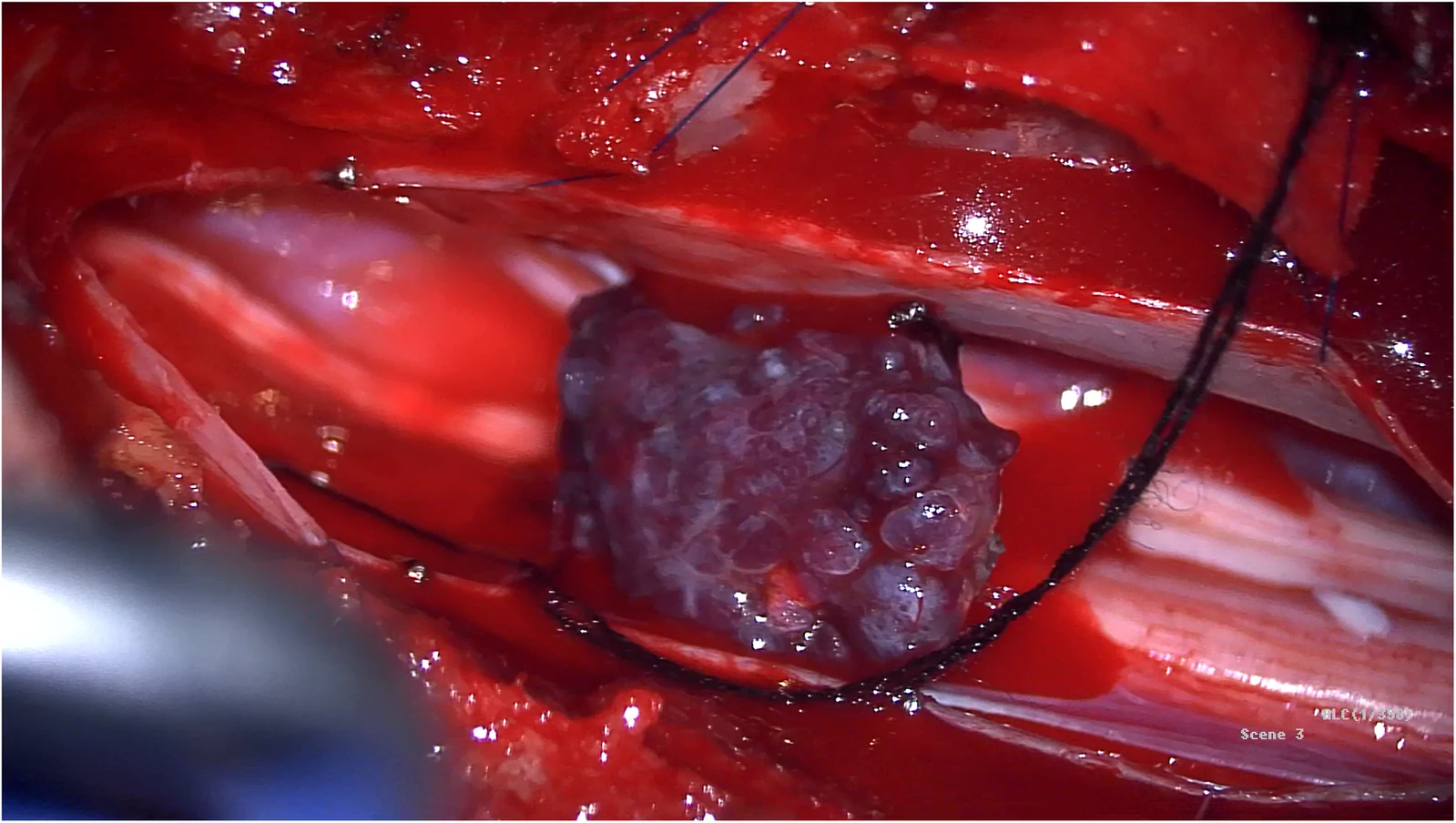
Intraoperative appearance. Intraoperative photograph showing a lobulated purple vascular lesion after dural opening, with adjacent neural structures and tortuous vessels on its surface.

Motor evoked potentials (MEPs) and somatosensory evoked potentials (SSEPs) were monitored and were reported to remain within normal limits throughout resection, without a documented alert. Original waveforms and numerical measurements, stimulation settings, and alert criteria were unavailable. The functional identity of the divided fascicles was not established. Hemostasis was secured, the subarachnoid space was irrigated, and the dura was closed tightly. Operative duration was 185 min. Estimated blood loss was 20 mL, and no transfusion was given. Meticulous hemostasis throughout the bilateral L1 laminectomy and microsurgical intradural dissection contributed to the low estimated blood loss.

### 2.3 Histopathological findings

The pathology report described a gray-black tissue nodule measuring 1.2 × 1.0 × 0.8 cm, with a loose, spongy cut surface and focal firmness. Microscopy showed variably sized thin-walled vascular channels lined by a single layer of flattened cells, with dilated blood-filled lumina and focal stromal fibrosis. The final diagnosis in the original pathology report was “cavernous hemangioma,” a term retained here to represent the source diagnosis accurately.

### 2.4 Postoperative course and outcomes

On postoperative day 1, the patient reported movement-related wound pain and had normal limb movement. The urinary catheter was removed by postoperative day 2. Bilateral hip pain and aching subsequently developed, at times radiating along the posterior aspects of both legs. Analgesic treatment and physical therapy were given, with progressive improvement. Early postoperative constipation was treated with lactulose.

On postoperative day 7, intravenous analgesia was no longer required. Mildly effortful urination was reported, without urinary frequency. Gross superficial sensation and pain perception were recorded as normal, and limb movement remained normal. The recorded oral analgesic regimen at that stage comprised tramadol 100 mg and pregabalin 150 mg twice daily. No postoperative residual urine measurement or urodynamic examination was available to establish whether the voiding complaint represented retention, altered coordination, or another mechanism.

Sutures were removed on postoperative day 9. At discharge on postoperative day 10, limb strength was grade 5, no superficial sensory deficit was recorded, and the wound had healed well. Discharge advice included a lumbar support for prolonged standing or sitting during the first month, analgesia and laxative treatment as needed, and lumbar MRI with contrast at three months. The record does not establish resolution of preoperative urgency or postoperative voiding difficulty. Follow-up lumbar MRI was obtained on 25 July 2025, 127 days after surgery (Figure 3). The available screenshots document postoperative imaging follow-up, but a formal radiology report and the complete image series were not available for assessment of residual disease. No contemporaneous clinical assessment of urinary or bowel function was available; radiological follow-up therefore cannot establish autonomic recovery.

**Figure 3:**
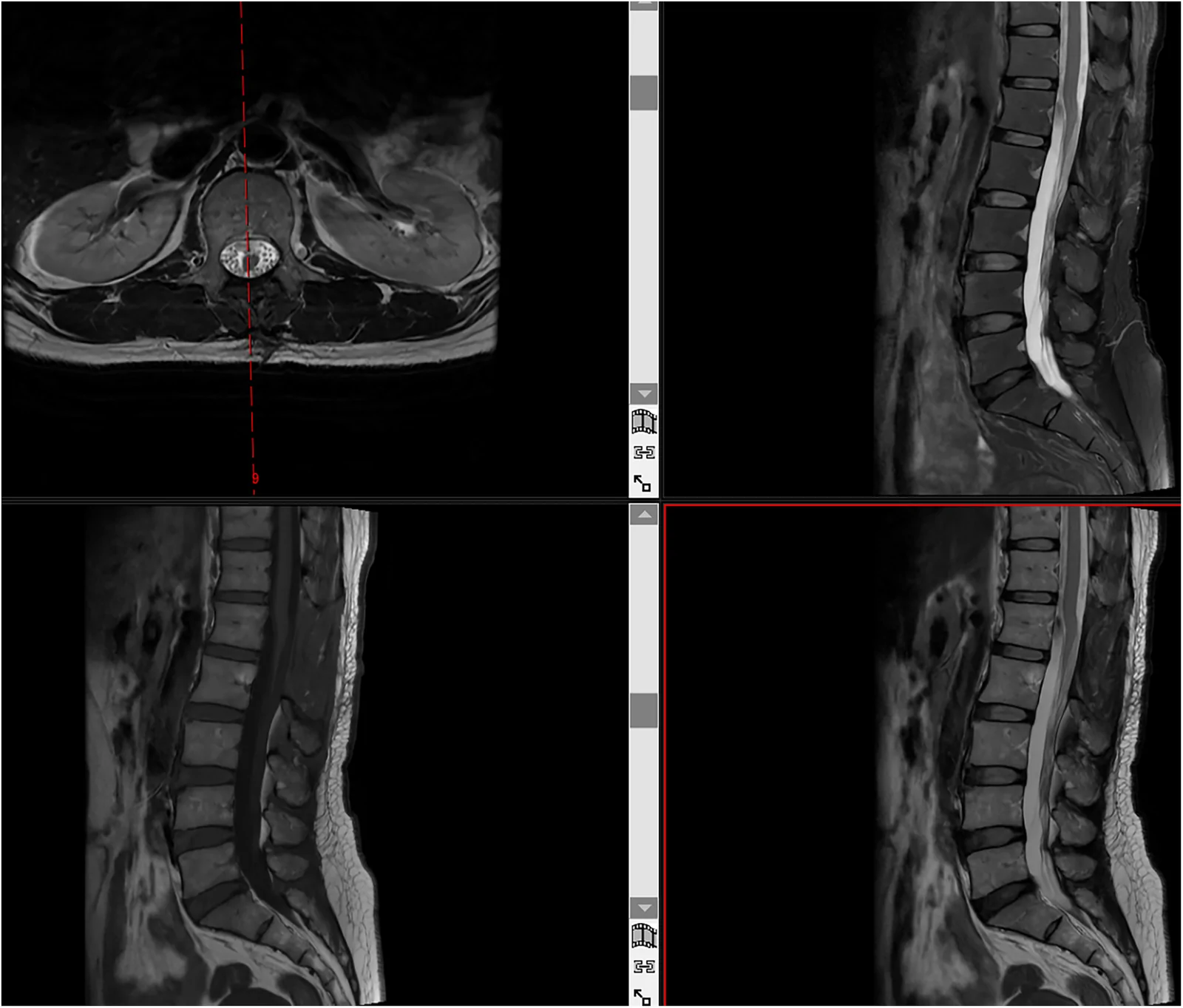
Postoperative lumbar MRI. Postoperative lumbar MRI obtained on 25 July 2025, four months after resection. The composite screenshot contains an axial view at the upper left and sagittal views in the remaining panels. No obvious residual mass is visible on the supplied screenshots; a formal report and the complete postoperative image series were unavailable for independent assessment.

## 3 Discussion

The principal lesson of this case is that minimal motor impairment can coexist with a symptomatic lesion involving the cauda equina. Urgency and distal sensory symptoms were the dominant preoperative complaints. They were clinically compatible with cauda equina involvement, but the available assessment did not prove that every symptom arose from the lesion. Concurrent degenerative findings and the absence of formal functional testing limit causal attribution. Likewise, preserved strength at discharge does not establish full autonomic recovery.

The L2 designation refers to the vertebral level of the intradural mass, not to an isolated L2 nerve-root lesion. Despite a left-sided imaging location, the toe symptoms were predominantly right-sided, with occasional similar symptoms on the left. Lumbosacral root bundles descend within the thecal sac at upper lumbar levels (6), so the vertebral location alone cannot determine the symptomatic root or side. The concurrent L5–S1 disc bulge is another possible contributor to distal toe symptoms, although its clinical significance is uncertain. Without root-specific functional mapping or selective diagnostic testing, the source of these distal symptoms cannot be established from this case.

The preoperative diagnostic evolution is also relevant. Although the admission diagnosis was schwannoma, the subsequent MRI report explicitly included cavernous hemangioma. It would therefore be inaccurate to describe this as a completely unsuspected vascular lesion. The combination of intrinsic T1 signal elevation, low heterogeneous T2 signal, and heterogeneous enhancement prompted consideration of blood products or proteinaceous material. Pathology confirmed the vascular diagnosis, but did not independently document repeated hemorrhagic episodes. The reported diffusion appearance was qualitative and should not be promoted as a validated discriminator.

We use “cavernous malformation” for the lesion in the clinical narrative. The 2025 International Society for the Study of Vascular Anomalies classification distinguishes vascular malformations from vascular tumors and discourages the historically ambiguous term “cavernous hemangioma” (7). We retain that term only when accurately conveying the wording of the original radiology or pathology report or cited literature. Its presence in the pathology report does not imply a separately diagnosed proliferative hemangioma.

Published experience supports this diagnostic overlap. Yang and colleagues found variable MRI signal and enhancement in 10 cauda equina cavernous angiomas, with gross total removal in all patients and improvement in nine over a mean follow-up of 41.1 months (1). Naseri and colleagues reported a lesion interpreted as schwannoma before pathological identification as cavernous hemangioma (2). These observations support including a vascular lesion in the differential, but do not establish a sufficiently specific imaging rule for individual patients.

In our patient, the operative challenge was a nerve bundle traversing the lesion rather than simple displacement of neighboring roots. Most fascicles were dissected free, but a small inseparable portion was divided. The operation therefore cannot be described as preserving every involved nerve fiber. The report by Apostolakis and colleagues provides a related example of root adherence necessitating sacrifice (3). Nogueira and colleagues also described a lesion involving cauda equina rootlets treated surgically (4). Such reports illustrate anatomical variability; they do not justify routine division of adherent fibers.

The reported stability of MEPs and SSEPs is reassuring only within the limits of those modalities. Without original waveforms, numerical measurements, stimulation settings, alert criteria, or root-specific functional mapping, this case cannot establish that the divided fascicles were nonfunctional or that sacral autonomic pathways were assessed. Bilateral radiating pain and mildly effortful urination developed during the postoperative period despite preserved limb strength. Their temporal relationship to surgery merits reporting, although the records cannot distinguish nerve manipulation, fascicular injury, medication effects, or other perioperative contributors.

Postoperative documentation would be more informative if it separately tracked pain, distal sensation, walking, urinary storage symptoms, urinary emptying, and bowel function. In this patient, a normal discharge motor examination and a healing incision captured only part of recovery. Repeat postvoid residual measurement and a dated account of urgency, voiding effort, continence, and bowel symptoms would clarify the clinical trajectory. Although MRI follow-up was completed approximately four months after surgery, a corresponding clinical assessment of autonomic function was not available.

The strength of this report is the concordance of contemporaneous clinical, operative, radiological-report, and pathology-report evidence, supplemented by preoperative and postoperative MRI screenshots and an intraoperative photograph. Limitations include a single retrospective case, unavailable original monitoring waveforms and numerical measurements, and undocumented stimulation settings and alert criteria, absent histological micrographs, an unavailable formal postoperative MRI report and complete imaging series, and detailed clinical follow-up restricted to hospitalization. The MRI obtained at 127 days does not establish longer-term urinary or bowel outcomes. Neither long-term autonomic recovery nor a general safety conclusion about fascicle sacrifice can be inferred. The literature discussion is focused and narrative, not a systematic review.

## 4 Conclusion

A cauda equina cavernous malformation should remain in the differential diagnosis of a symptomatic L2 intradural extramedullary mass. This case documents complete excision as assessed intraoperatively after separation of most involved fascicles and division of a small inseparable portion. Preserved discharge strength coexisted with postoperative pain and a documented voiding complaint. The case supports careful reporting of nerve involvement and domain-specific outcomes, while longer follow-up is needed to assess autonomic recovery and recurrence.

## Data Availability

The original contributions presented in the study are included in the article/Supplementary Material, further inquiries can be directed to the corresponding author.
